# Surface plasmon resonance imaging of pathogens: the *Yersinia pestis* paradigm

**DOI:** 10.1186/s13104-015-1236-3

**Published:** 2015-06-24

**Authors:** Hong T T Huynh, Guillaume Gotthard, Jérome Terras, Gérard Aboudharam, Michel Drancourt, Eric Chabrière

**Affiliations:** Faculté de médecine, Unité de Recherche sur les Maladies Infectieuses et Tropicales Emergentes (URMITE), UMR CNRS 7278, IRD 198, INSERM 1095, 27, Boulevard Jean Moulin-Cedex 5, Marseille, France

**Keywords:** Surface plasmon resonance imaging, *Yersinia pestis*, Plague, Detection

## Abstract

**Background:**

*Yersinia pestis*, causing deadly plague, is classified as a group A bioterrorism bacterium. Some recent DNA-based methods were used for detection of bioterrorism agents.

**Results:**

*Y. pestis* was used as a model organism to develop an immunosensor based on surface plasmon resonance imaging (SPRi) using monoclonal antibody against *Y. pestis* F1 antigen. The experimental approach included step-by-step detection of *Y. pestis* membrane proteins, lysed bacteria, intact bacteria, mock-infected powder and mock-infected clinical specimens. SPRi detected on average 10^6^ intact *Y. pestis* organisms in buffer, in mock-infected powder and in a 1:4 mixture with HEL cells.

**Conclusions:**

This study offers the proof-of-concept of the SPRi-based detection of a human pathogen in both environmental and clinical specimens.

## Background

Plague is a deadly zoonosis caused by the bacterium *Yersinia pestis* [1]. It remains a public health problem in many tropical countries including subtropical African countries [1, 2] and it is re-emerging in North Africa [3, 4]. There are no longer any plague foci in Europe, though plague had caused devastating epidemics for two millennia [1]. Also, *Y. pestis* has been classified as a group A bioterrorism agent [5]. Currently, the detection of *Y. pestis* in environmental and clinical specimens, is based on the culture of *Y. pestis*, a process requiring at least 24 h and a biosafety level 3 laboratory (BSL3) [6]; and PCR-based detection of DNA sequences [7], whose specificity has recently been challenged by the observation of *pla* sequences, thought to be *Y. pestis*-specific, in other organisms and non-infected rodents [8]. Also, immunochromatography detection of the *Y. pestis*-specific F1 antigen [9–11] is used for research, as this diagnostic assay is not widely available. Recently, some new approaches such as high-throughput RT-PCR-coupled ESI–MS assay and Luminex were applied to the detection of bioterrorism agents [12, 13]. These techniques required principally amplified DNA and apparently remained time-consuming.

In this study, *Y. pestis* was used as a model organism to test whether surface plasmon resonance imaging (SPRi) could be used as a novel technique for the rapid detection of pathogens in environmental and clinical specimens. SPRi has advantages (high-throughput, real time, label-free, multi-detection and sensitive) which could be applied to the detection of organisms, such as *Y. pestis*. Several studies of immune reactions (cells—antibodies, peptides—antibodies) have been conducted with this technique [14, 15]. Recently, this technique was used to detect the plant pathogenic bacterium *Acidovorax avenae* subsp. *citrulli* [16]. However, until now, SPRi has not been used to detect human pathogenic bacteria.

In this study, we challenged the proof-of-concept that SPRi could be used for the rapid detection of highly pathogenic organisms in environmental and clinical specimens, using *Y.* *pestis* as a model organism. We developed a step-by-step experimental approach to test membrane proteins, lysed bacteria, intact bacteria (*Y. pestis* Orientalis YPA, Medievalis 6B4), mock-infected powder and mock-infected clinical specimens.

## Methods

### Materials and instruments

CS-SPRi Biochips and CS-SPRi Slides covered by a thin layer of gold and functionalized NHS groups were purchased from HORIBA (Palaiseau, France). The ligand used in this study was a mouse monoclonal antibody (mAb) against the F1 antigen of *Y. pestis* [YPF19] (4.3 mg/mL) purchased from GenWay Biotech, Inc. (Gentaur, Belgium). A mouse non-immune control serum was produced and purified in our laboratory (URMITE, Marseille, France). The protocol to collect serum from non-immune mice has been approved by the French National Ethic Committee for Animals under the reference number 60-12112012. Sodium acetate, ethanolamine and glycine were purchased from Sigma-Aldrich (Saint-Quentin Fallavier, France), while phosphate buffered saline (PBS) was obtained from bioMérieux (La Balme-les-Grottes, France).

### Ligand immobilisation

Ligands diluted in 10 mM sodium acetate, pH 5 at different concentrations (mAb: 1, 0.5, 0.25 mg/mL; control serum: 1 mg/mL) were automatically deposited onto the chip (6 spots for each ligand with a distance of 0.7 mm between each spot) using a 300 nm diameter ceramic needle controlled by the mechanical SPRi-Arrayer (HORIBA, Palaiseau, France). Needle rinsing with distilled water for 3 s, followed by drying with compressed air for 3 s, were automatically repeated 3 times both before and after each ligand was deposited. The antibody was immobilised at room temperature in a humid chamber set to 60% relative humidity. The chip was air-dried and placed in the chip box at 4°C until use.

### Analyte preparation

#### Membrane proteins

Suspensions of *Y. pestis* strain YPA (an Orientalis biotype, CSUR P100) in PBS were sonicated 5 times for 1 min on ice at an amplitude of 30 W with Q700 Sonicator (Qsonica, LLC, DENTA LABO, Avignon, France). The tubes were centrifuged for 5 min at 4.000×*g*. Supernatant was ultra-centrifuged for 1 h at 100.000×*g*. The pellet containing membrane proteins was suspended in 500 µL PBS or 500 µL 0.2% Triton X-100, 30 mM Tris HCl pH 8 and 2 mM MgCl_2_ and was then left overnight at 4°C to solubilize the membrane proteins. Following the same procedure, *Escherichia coli* was used as a negative control.

#### Lysed bacteria

Five hundred µL of various concentrations of *Y. pestis* YPA were broken with acid-washed glass beads in a screw-cap tube using a FastPrep^®^-24 Instrument (MP Biomedicals, Illkirch, France) at a speed 4.0 m/V for 40 s. The tube was then centrifuged for 30 s at 6.700×*g* and the supernatant was analysed with SPRi. *Bartonella quintana*, prepared according to the same protocol, was used as a negative control.

#### Intact bacteria

Virulent *Y. pestis* YPA and *Y. pestis* Medievalis 6B4 were cultured on Columbia agar and 5% sheep blood (bioMérieux) at 32°C, 5% CO_2_ for 3–5 days. *E. coli* and *Staphylococcus aureus* used as negative controls were cultured in the same medium at 37°C. Virulent *Y. pestis* was handled in a BSL3. Bacteria were inactivated with 70% ethanol. The SPRi specificity test was carried out with *Y. pestis* YPA, *Y. pestis* Medievalis, *E. coli* and *S. aureus*. SPRi sensitivity was tested with different concentrations of *Y. pestis* YPA.

#### Sandwich test

A “sandwich” test (mAb/*Y. pestis*/mAb) was developed on SPRi in order to enhance the sensitivity of the SPRi assay. On a chip with 1 mg/mL immobilized mAb, *Y. pestis* YPA (1.2 × 10^1^ to 1.2 × 10^7^ CFU/mL) was tested within 10 min, followed by an injection of mAb of 1/500. The area under the curve for each injection was analysed using GraphPad PRISM V6 software (GraphPad Software, Inc., USA). The first phase (bacterial injection) from 0 to 11.5 min, the second phase (antibody injection) from 11.5 to 22 min and the entire process from 0 to 22 min were analysed.

#### Mock-infected powder

*Y. pestis* YPA mixed at different concentrations (10^8^, 10^6^, 10^4^ CFU/mL) with flour powder was tested on SPRi to estimate whether this technique could detect the pathogen in environmental samples in mimicking a bioterrorist alert. Powder mixed with either PBS or *E.* *coli* were used as negative controls. The experiment was repeated three times.

#### Mock-infected clinical specimens

*Y. pestis* YPA mixed with HEL cells at ratio 1:1, 1:10, 1:100 was used as a model to evaluate the capability of SPRi to detect the pathogen in infected clinical specimens. A suspension of non-infected HEL cells was used as negative control. This experiment was performed in triplicate.

### SPRi experiments

The experiments were conducted using the SPRi-Plex II system and monitored using SPRi P5.0.2-View software (HORIBA, Palaiseau, France). The running buffer for the SPRi-Plex II system was 10 mM PBS. Initial buffer flow rate was 500–1,000 µL/min to fill the fluid system for 15 min. Once the chip was inserted into the machine, the analysis cell was filled at a flow rate of 750–1,000 µL/min, followed by a flow rate of 50 µL/min and a temperature of 37°C was used for all experiments. After system stabilization, plasmon images were acquired by software and system mirror and camera. The study area deposits were detected on the previously acquired high-contrast image and spot and spot family definitions were performed. The plasmon curve and resonance angle were determined for each spot. The mirror system was shifted by the resonance angle and the experiments were conducted with this value.

The surface of the chip was saturated for 10 min with 500 µL of 1 M ethanolamine pH 9 and regenerated for 10 min with 500 µL of 10 mM glycine pH 1.85 to remove any non-covalent bindings (unfixed antibodies and ethanolamine). The system was calibrated by comparing the reflectivity of 12.5 mM PBS and of 10 mM PBS buffer. Control serum not targeting *Y. pestis* was defined as a reference surface. This was used to make subtracted curves of each spot family in real time to eliminate non-specific signals. A 400 µL-volume of each sample was loaded into the system (200 µL for analysis, 200 µL for the carrier fluid). The interaction of each sample with the surface of the chip was measured for 10 min. Changes in reflectivity were monitored in real time on the graph and on the chip image. After each experiment, the chip was regenerated with 10 mM glycine pH 1.85.

### Data analyses

The data were analysed using SPRi-Analysis software V1.2. The reflectivity change subtracted from the negative control plus two standard deviations was considered as positive. The data were analysed by means of a t-test in GraphPad PRISM V6 for p value (GraphPad Software, Inc., USA).

## Results

### Tests with membrane proteins

**Figure 1:**
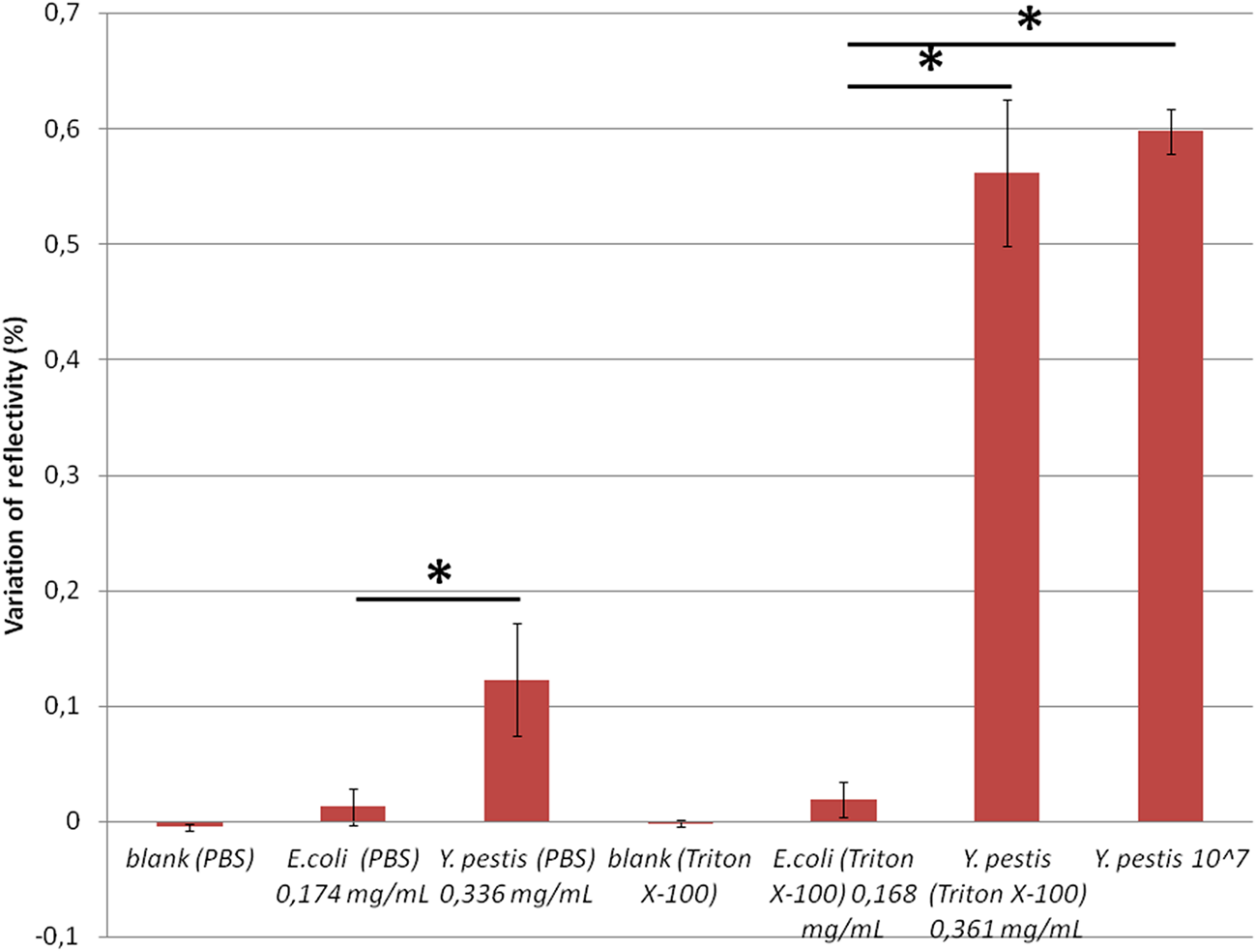
Test of membrane proteins with SPRi. Membrane proteins of *E. coli* (negative control) and *Y. pestis* solubilized in PBS and in Triton X-100 were tested. mAb concentration was 1 mg/mL. *Asterisk* represents a statistically significant difference with *E. coli* solubilized in PBS and in Triton X-100.

In the first series of SPRi experiments, we tested the membrane proteins of *Y. pestis* YPA and *E. coli* (negative control) solubilized in PBS and in Triton X-100. As expected, in all cases the membrane proteins of *E.* *coli* gave the same signals as the blank (Figure 1). The membrane proteins of *Y. pestis* YPA solubilized in PBS (0.336 mg/mL) and in Triton X-100 (0.361 mg/mL) gave statistically different signals to that of *E. coli* solubilized in PBS (0.174 mg/mL) and in Triton X-100 (0.168 mg/mL) (p < 0.05). The detection signals of *Y.* *pestis* proteins solubilized in Triton X-100 was higher than in PBS (p < 0.05). Indeed, SPRi was able to detect *Y. pestis* membrane proteins extracted with PBS and even more so with Triton X-100.

### Sensitivity with lysed and intact bacteria

**Figure 2:**
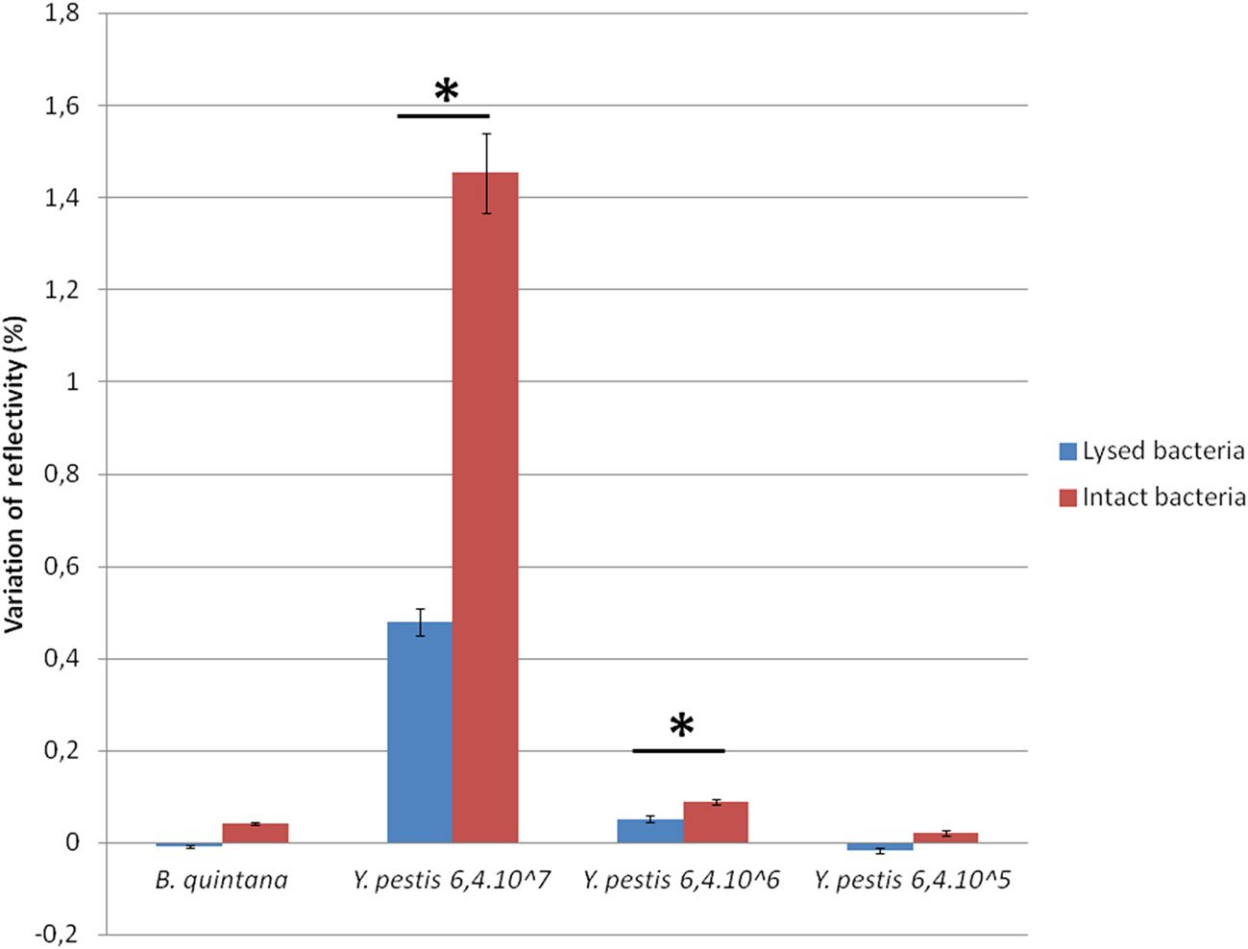
Comparison of the sensitivity of SPRi in detection of lysed bacteria (FastPrep) and intact bacteria. The signals with lysed bacteria were less statistically significant than intact bacteria, whatever the concentration. mAb concentration was 1 mg/mL. *Asterisk* represents a statistically significant difference between lysed and intact bacteria.

SPRi experiments were conducted with decreasing concentrations of lysed *Y. pestis* and lysed *B.* *quintana* used as negative control according to the FastPrep protocol to test detection sensitivity of lysed bacteria. Figure 2 shows that, as expected, the negative control remained negative. SPRi was able to give statistically different signals up to 6.4 × 10^6^ CFU/mL lysed *Y.* *pestis* compared to the negative control (p < 0.05). The same experiment performed with intact bacteria gave the same detection threshold (6.4.10^6^ CFU/mL). Figure 2 also shows that the signals with lysed bacteria were statistically less significant than intact bacteria, whatever the concentration (p < 0.05).

### Specificity and effect of mAb concentration

**Figure 3:**
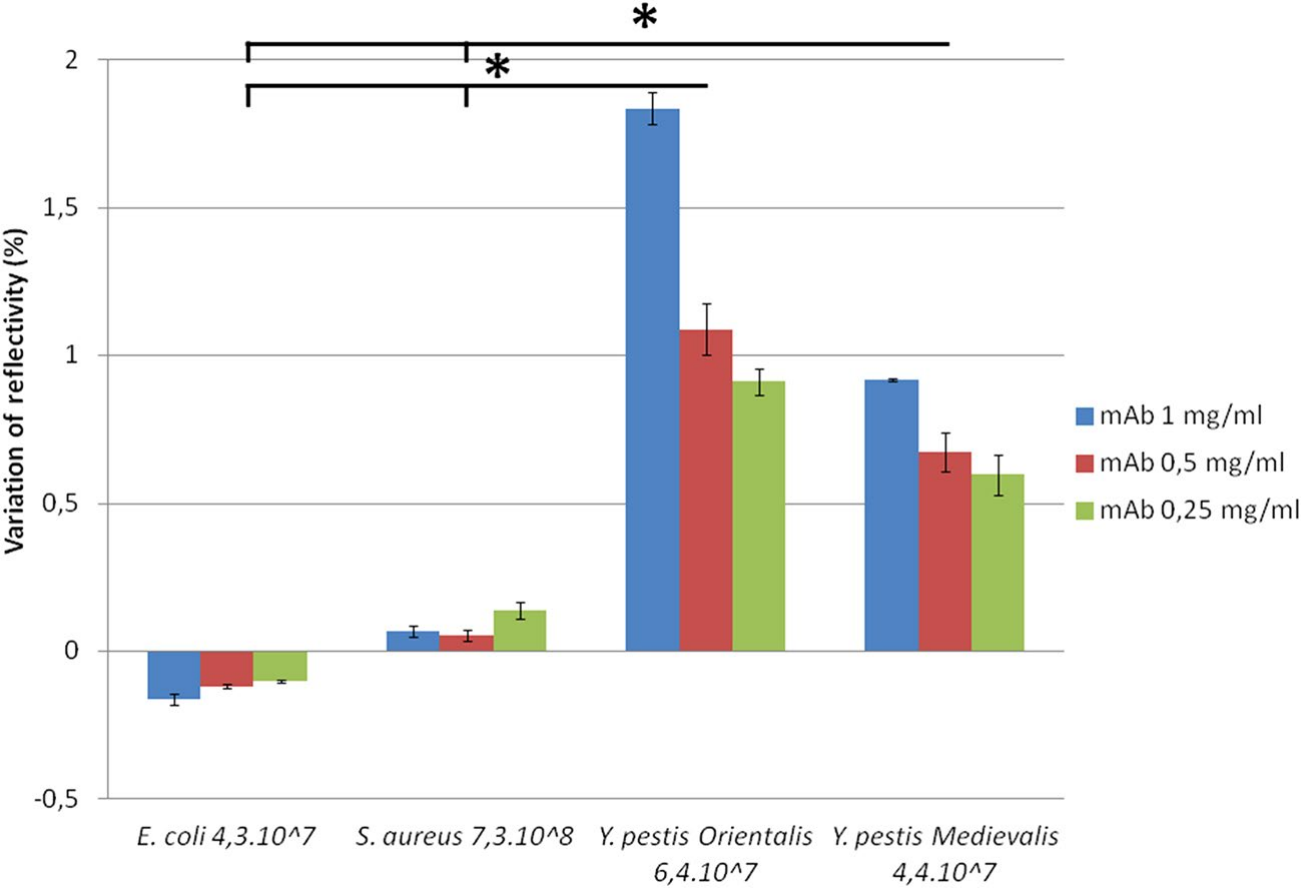
Specificity tests of SPRi with intact bacteria. *Asterisk* represents a statistically significant difference with *E. coli* and *S. aureus*.

In this step, we wanted to test the specificity of this technique. Four different kinds of intact bacteria (*E. coli*, *S. aureus*, *Y. pestis* YPA and *Y. pestis* 6B4) were tested with anti-*Y. pestis* mAb previously immobilized on a chip at 1, 0.5 and 0.25 mg/mL. In Figure 3, *E. coli* and *S.* *aureus* did not show any significant interaction with anti-*Y. pestis* mAb, as expected. *Y.* *pestis* YPA and *Y. pestis* 6B4 gave significantly different signals with mAb, whatever the mAb concentration, compared *E. coli*, *S. aureus* (p < 0.05). Moreover, we noted that interaction between mAb and *Y. pestis* correlated with mAb concentration. mAb at 1 mg/mL gave statistically significant signals compared to 0.5 mg/mL and 0.25 mg/mL in the test with the same concentration of *Y. pestis* (p < 0.05).

### Sandwich test

**Figure 4:**
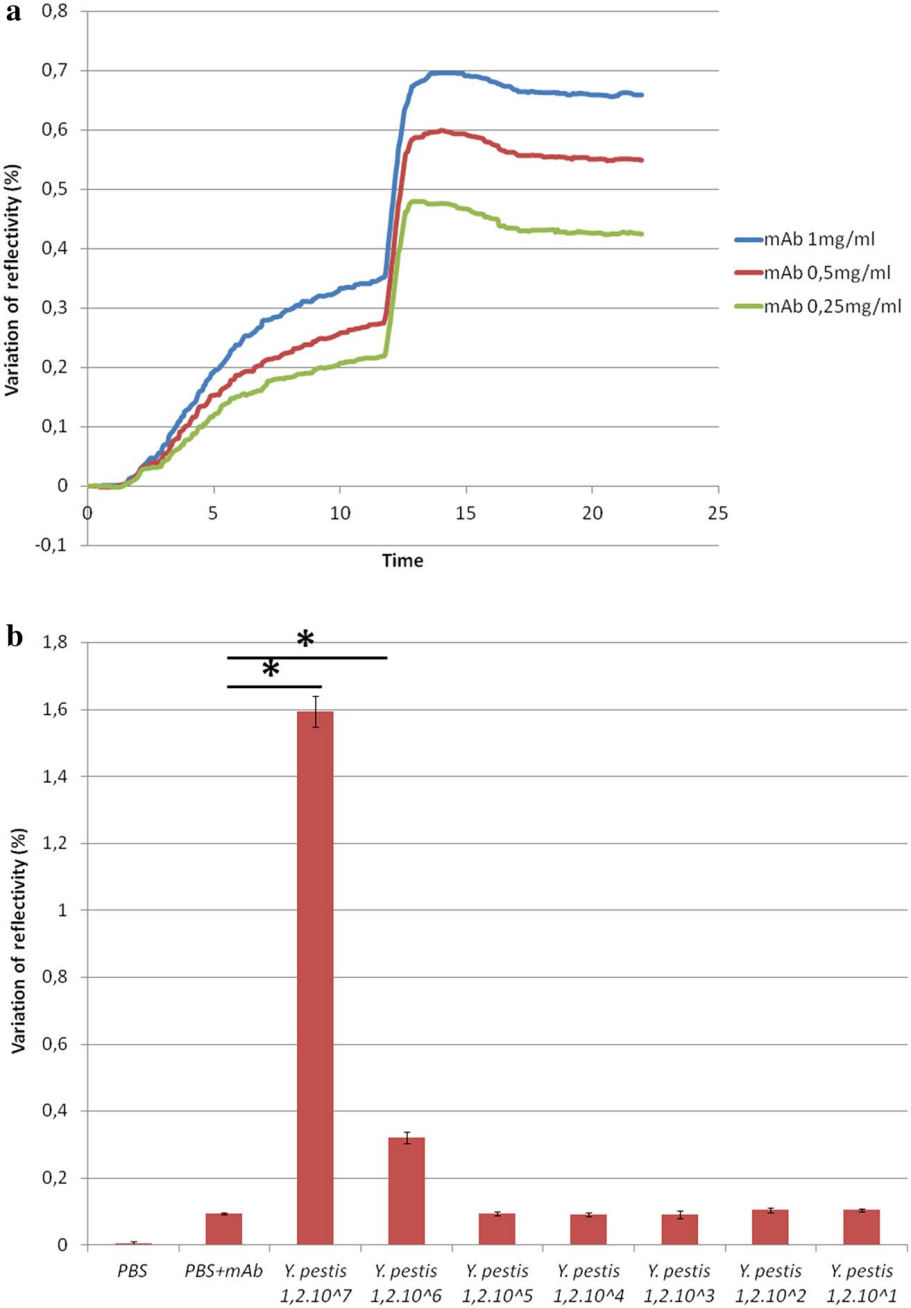
**a** Area under t he reflectivity curve. The second phase area (injection of mAb, 12–22 min) was 4 times greater than the first phase (injection of bacteria, the conventional technique, 0–12 min), respectively. **b** Sandwich tests (mAb + bacteria + mAb). Decreasing concentrations of *Y. pestis* (CFU/mL) were tested by the sandwich technique, PBS + mAb was used as negative control. mAb concentration was 1 mg/mL. *Asterisk* represents a statistically significant difference with PBS + mAb.

In an effort to improve the sensitivity of SPRi in detection of bacteria, we applied a sandwich technique (mAb/*Y. pestis*/mAb). The sandwich test signals were significantly higher than those obtained following non-specific binding of the injection of control (PBS and monoclonal antibody). In Figure 4a, the analysis of the area under the curve showed that the sandwich test could improve the sensitivity of SPRi. In Figure 4b, by testing decreasing concentrations of bacteria (1.2 × 10^7^ to 1.2 × 10^1^ CFU/mL) using the sandwich technique, SPRi was able to detect *Y. pestis* YPA up to 1.2 × 10^6^ CFU/mL (p < 0.05).

### Mock-infected powder and clinical specimens

**Figure 5:**
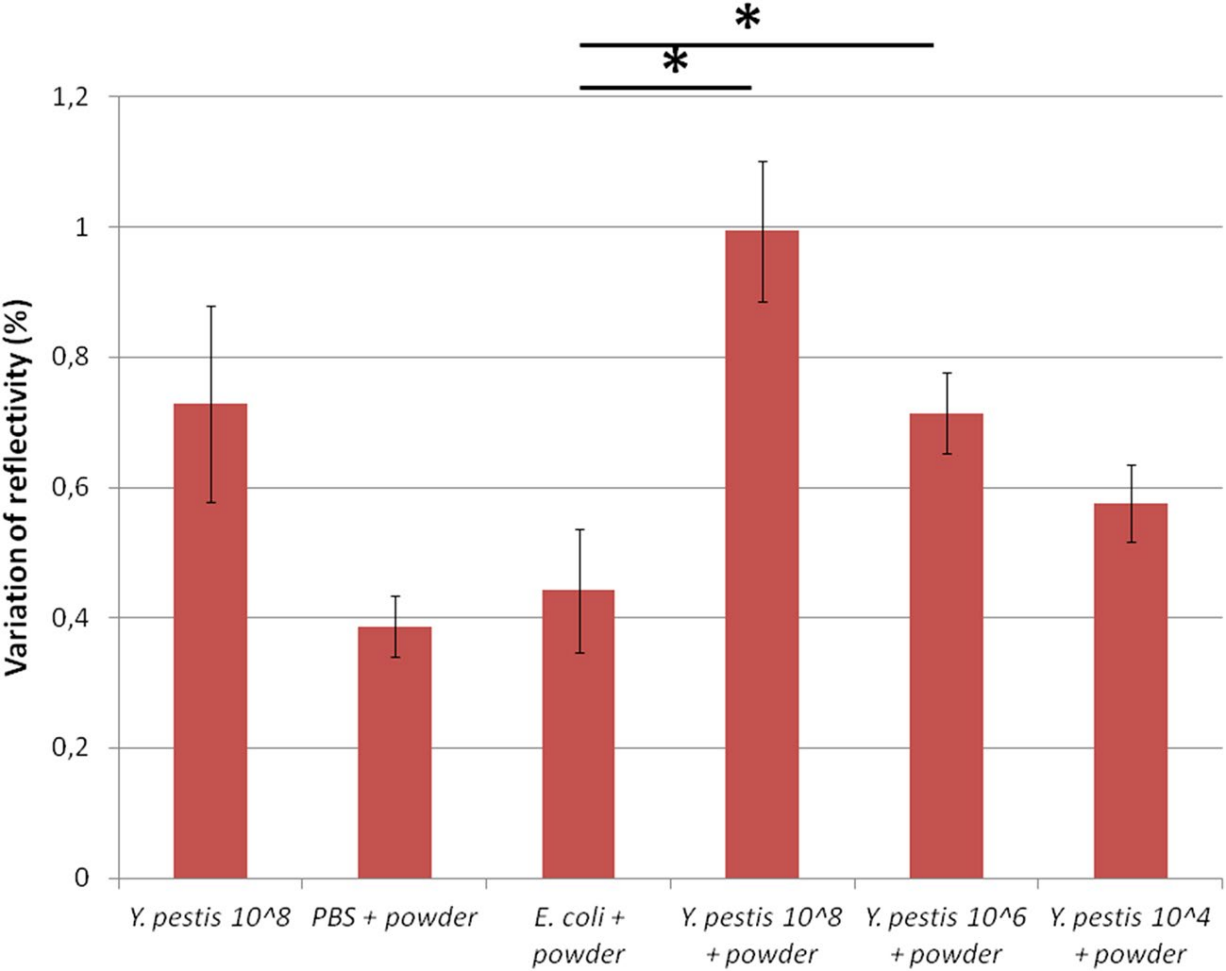
Test of mock-infected powder with SPRi. mAb concentration was 1 mg/mL. *Asterisk* represents a statistically significant difference with *E. coli* + powder.

**Figure 6:**
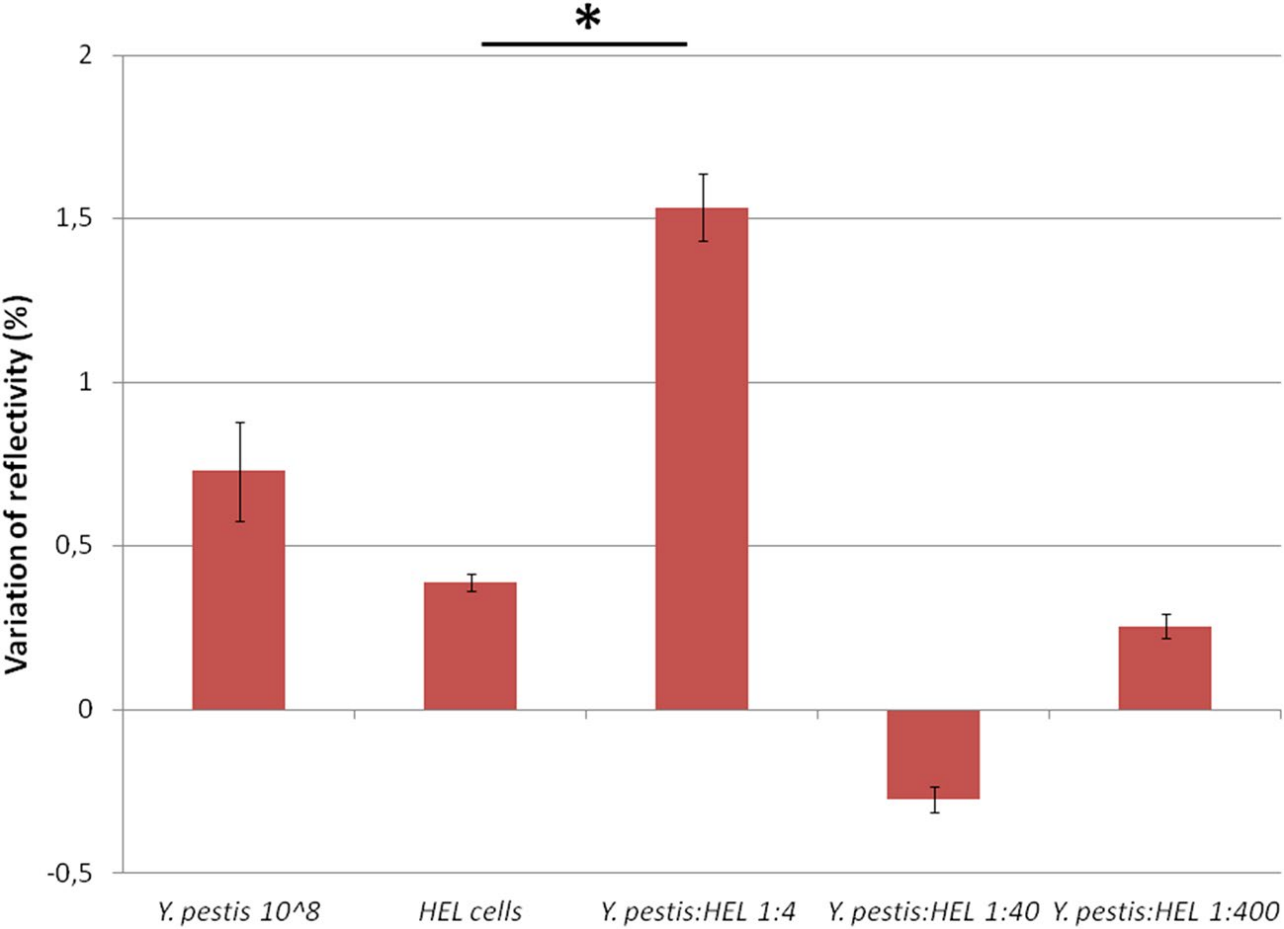
Test of mock-infected clinical specimens with SPRi. mAb concentration was 1 mg/mL. *Asterisk* represents a statistically significant difference with HEL cells.

To evaluate the ability to detect pathogen in environmental and clinical specimens, we tested *Y. pestis* mixed with powder and HEL cells. Figure 5 shows that mixtures of *Y. pestis* YPA at 10^8^ and 10^6^ CFU/mL with powder gave statistically significant signals compared to the negative controls (PBS or *E. coli* with powder) (p < 0.05). In Figure 6, *Y. pestis* YPA (10^6^ CFU/mL) could be detected in a mixture with HEL cells at a cell number ratio of 1:1, corresponding to a mass ratio of 1:4 (p < 0.05). In this experiment, the protein concentrations of *Y. pestis* YPA and HEL were 0.61 and 2.3 mg/mL, respectively. The other mixtures did not give statistically significant differences in refractive index by comparison with the negative control (non-infected cells). Here, a mixture of 10^6^ CFU/mL of *Y. pestis* with powder or with HEL cells could be also detected with SPRi.

## Discussion

Rapid and accurate detection of harmful organisms in both clinical and environmental specimens is a constant goal to serve medical diagnosis and protection. SPRi, being reported as a high-throughput method for the detection of molecule interactions, including antigen–antibody interactions, held promise to help in the rapid detection of organisms. The results reported in the present study were validated by numerous controls, indicating that SPRi yielded the specific detection of *Y. pestis* used as a model organism.

As SPRi-based measures incorporate no-labelled Mab and since all substances could affect the reflectivity index of the solution, it was important to distinguish specific interactions from non-specific ones. To eliminate non-specific binding to antibodies immobilized on the chip, the antibody not targeting the bacteria could be used as a blank [17]. We used the non-immune purified serum as a reference surface and the injection of other bacteria as negative controls. Reference surface interactions were automatically subtracted and results compared with the negative control to identify the specific interactions.

Moreover, specificity was confirmed by the observation that Gram-negative (*E. coli*, *B.* *quintana*) and Gram-positive (*S. aureus*) bacteria did not give any signal with anti-*Y. pestis* mAb. Moreover, signal intensity increased with antibody concentration, as has previously been reported [16]. Here, 6.4.10^6^/mL lysed or intact bacteria alone or in mock-infected powder and clinical specimens could be detected by SPRi. SPRi was significantly less sensitive when lysed bacteria were used rather than intact bacteria, by contrast with a previous observation involving *A. avenae* subsp. *citrulli* [16]. The sandwich assay, however, was able to 2.4.10^5^*Y. pestis* bacteria, in line with a previous study by Puttharugsa et al. [16].

Altogether, SPRi was able to detect 10^5^*Y. pestis* bacteria, an inoculum in the range of that expected to cause deadly plague after natural or criminal exposure to the pathogen. Indeed, the lowest inoculum of *Y. pestis* that consistently gave a 100% mortality rate in a mouse model was 10^4^ CFU [18], suggesting that an inoculum of bacteria found in a bioterrorism attack would be >10^4^ CFU. Specimen incubation for a few hours prior to the SPRi assay is a promising way to further improve sensitivity, as previously reported for *E.* *coli* O157:H7 [19]. Some new approaches were applied in the detection of bioterrorism agents. These techniques were principally based on amplified DNA-sequence detection including high-throughput RT-PCR-coupled ESI–MS assay and Luminex assay [12, 13]. According to the current state of the art, SPRi is less sensitive than PCR-based techniques [20] and this sensitivity remains to be improved. Improving SPRi sensitivity is warranted as SPRi has advantages over PCR-based techniques: SPRi is specific whereas it has been shown that *pla*, a long-standing target for the PCR-based detection of *Y. pestis* is in fact detectable in non-Yersinia organisms, including the host [8]. In particular, *Y. pestis* is easily engineered to intentionally modify PCR targets, thus helping the pathogen and bioterrorism agent escape detection. Also, SPRi is not too time-consuming, requiring only 40 min for a direct assay (20 min for infection of analyte, 20 min for negative control) and 1 h for a sandwich assay. A chip with many kinds of mAb could be used to detect different organisms. Moreover, this chip could be reused several times. This makes SPRi appropriate for rapid, cheap, multiplexed detection. Once sensitivity is improved, this technique would be perfectly suitable for sample analysis in the context of a bioterrorism emergency or for routine analysis in an epidemic area.

## Conclusions

In conclusion, SPRi is a new technique for the rapid detection of bacteria in environmental and clinical specimens, as illustrated here using *Y. pestis* as a model organism. Future improvements will be directed towards increasing the sensitivity of the technique.

## Abbreviations

SPRi: surface plasmon resonance imaging
mAb: monoclonal antibody
PBS: phosphate buffered saline
CFU: colony-forming unit
HEL: human embryonic lung cells
BSL3: biosafety level 3 laboratory
PCR: polymerase chain reaction
